# Gut microbiome of endangered *Tor putitora* (Ham.) as a reservoir of antibiotic resistance genes and pathogens associated with fish health

**DOI:** 10.1186/s12866-020-01911-7

**Published:** 2020-08-12

**Authors:** Himani Khurana, Durgesh Narain Singh, Anoop Singh, Yogendra Singh, Rup Lal, Ram Krishan Negi

**Affiliations:** 1 Fish Molecular Biology Laboratory, Department of Zoology, University of Delhi, Delhi, 110007 India; 2 Molecular Biology Laboratory, Department of Zoology, University of Delhi, Delhi, 110007 India; 3 Laboratory of Microbial Pathogenesis, Department of Zoology, University of Delhi, Delhi, 110007 India; 4 Present address: The Energy and Resources Institute Darbari Seth Block, IHC Complex, Lodhi Road, New Delhi, 110003 India

**Keywords:** Gut microbiome, Endangered *Tor putitora*, Virulence genes, Antibiotic resistance genes, Opportunistic pathogens

## Abstract

**Background:**

*Tor putitora*, the largest freshwater fish of the Indian subcontinent, is an endangered species. Several factors have been attributed towards its continuous population decrease, but very little is known about the gut microbiome of this fish. Also, the fish gut microbiome serves as a reservoir of virulence factors and antibiotic resistance determinants. Therefore, the shotgun metagenomic approach was employed to investigate the taxonomic composition and functional potential of microbial communities present in the gut of *Tor putitora*, as well as the detection of virulence and antibiotic resistance genes in the microbiome*.*

**Results:**

The analysis of bacterial diversity showed that *Proteobacteria* was predominant phylum, followed by *Chloroflexi*, *Bacteroidetes,* and *Actinobacteria*. Within *Proteobacteria*, *Aeromonas* and *Caulobacter* were chiefly present; also, *Klebsiella, Escherichia,* and plant symbionts were noticeably detected. Functional characterization of gut microbes endowed the virulence determinants, while surveillance of antibiotic resistance genes showed the dominance of β-lactamase variants. The antibiotic-resistant *Klebsiella pneumoniae* and *Escherichia coli* pathovars were also detected. Microbial genome reconstruction and comparative genomics confirmed the presence of *Aeromonads*, the predominant fish pathogens*.*

**Conclusions:**

Gut microbiome of endangered *Tor putitora* consisted of both commensals and opportunistic pathogens, implying that factors adversely affecting the non-pathogenic population would allow colonization and proliferation of pathogens causing diseased state in asymptomatic *Tor putitora.* The presence of virulence factors and antibiotic resistance genes suggested the potential risk of dissemination to other bacteria due to horizontal gene transfer, thereby posing a threat to fish and human health. The preservation of healthy gut microflora and limited use of antibiotics are some of the prerequisites for the conservation of this imperilled species.

## Background

The gastrointestinal tract is a complex environment inhabited by diverse groups of microbial communities [1–3]. The gut bacteria play an important role in maintaining the normal physiology, nutrition, health, homeostasis, protection against pathogens, and functioning of the host immune system [2, 4–7]. There has been a growing interest in understanding the composition of symbiotic and pathogenic bacteria in the gut [6]. The symbiotic bacteria present in the gut provide several benefits to their hosts, such as digestion of complex indigestible food materials, production of important secondary metabolites, and defence against pathogens [2, 7–10], several opportunistic bacterial pathogens are also reported in the gut microbial community [11, 12]. Alterations in normal gut microflora reduce the competition for pathogens and result in their overgrowth leading to the diseased state [13]. Microbial dysbiosis causes impairment of the normal activity of digestive enzymes, damage to gut tissue, and increased infiltration of opportunistic pathogens and toxicants [14]. This fact has drawn considerable attention to find out potential probiotic, symbiotic, and pathogenic bacteria that may have a profound influence on the host physiology and health. While there are several studies on the fish gut microbiome, hitherto, there are gaps in our understanding on the structure and function of the gut microbiome in endangered fish [1, 2, 15, 16].

Most of the knowledge with respect to the fish gut microbiome was based on the use of culture-based methods for the investigation of microbial communities [6, 17]. However, culture-independent methods allow identification of a large proportion of microbial diversity than could be observed with culture-based studies [18]. Although 16S rRNA (marker-gene) sequencing provides information about the taxonomic composition of microbial communities, it provides limited information about their functional capabilities and metabolic pathways [19]. On the other hand, whole genome shotgun metagenomics can overcome these caveats and allow for a deeper understanding of the gut microbial communities and host-microbiota interactions [19–21]. The composition of microbial communities residing in the intestinal tract of different fish showed the presence of both beneficial and pathogenic microbes [2, 22–25]. However, it remains a challenge to elucidate whether the gut microflora drives for protection (friends) or disease development (foes) in fish. The fish gut is natural reservoir of *Aeromonas*, *Pseudomonas*, *Vibrio*, *Streptococcus,* and other coliforms [26–30]. The presence of virulence genes and antibiotic-resistance genes in *Aeromonas* spp. from freshwater may also be responsible for causing infections in humans as there have been reports of transmission of infectious *Aeromonas* from fish following injuries during handling*,* practicing aquaculture systems or pet fish keeping [31–34]. *Aeromonads*, the predominant species associated with the gastrointestinal tract of aquatic animals, are known to cause a multitude of diseases in freshwater fish [7, 30, 35, 36]. Among all the *Aeromonads*, *Aeromonas veronii* has the greatest range in virulence and has been associated with infectious abdominal dropsy in fish [37]. Also, there have been reports on the presence/transmission of opportunistic and other pathogenic bacteria in fish, making them potential carriers [26, 27, 29, 38–41]. Thus, it is imperative to detect virulence genes in the microbiome of fish for their possible transmission.

The aquaculture sector is a major contributor to the world’s production for food [22]. Overuse of antibiotics in aquaculture has led to the rapid emergence of antibiotic resistance genes (ARGs) in the aquatic environment where fish serve as a reservoir of multidrug-resistant bacteria and their potential mobilization [19, 42–44]. The dissemination of antibiotic resistance genes from fish bacteria to human pathogens is a serious threat to public health [43, 44]. Surveillance of ARGs in fish would aid in the development of regulations for the application of antibiotics in aquaculture.

*Tor putitora* (Ham.) is commonly known as golden mahseer due to its large size, attractive golden colour and sport values. It has been promoted as a ‘flagship’ species of the Indian subcontinent [45, 46] and has been enlisted endangered by International Union for Conservation of Nature (2010) and IUCN Red List assessment [47]. It is widely distributed in Afghanistan, Bangladesh, Bhutan, Myanmar, Nepal, Pakistan, and in India, its distributional range is in the Northeast Himalaya which includes Himalayan foothills, Garo hills of Meghalaya and Challou river of Manipur [45]. It is an indigenous fish species and forms mainstay fishery of upland Himalayan region [48]. It is consumed by people because it contains high-quality proteins making it a potential resource for aquaculture industries. Besides this, it is also a good source of minerals acting as diet supplements [48]. *Tor putitora* is an economically important fish because it has great culinary value, forms lucrative sport fishery in the Himalayan river, and provides employment opportunities to locals [49]. Despite this, very limited research efforts have been made to investigate the commensal/beneficial/pathogenic bacteria in the gut of this fish species [39]. In India, the Gobindsagar reservoir (31°24′59.99″ N, 76°29′59.99″ E) is one of the largest man-made lakes which port *Tor putitora* [50]. Mahseer used to constitute as high as 9% of the total catch during 1984–85, which has decreased rapidly to 1% during 1999–2000 [50]. Therefore, the major objective of this study was to understand the taxonomic composition and functions of bacterial communities associated with the gut of *Tor putitora*. The second focus of this study was to understand the antibiotic resistance pattern of gut bacterial communities.

## Results

### Taxonomic composition of fish gut microbiome

**Fig. 1:**
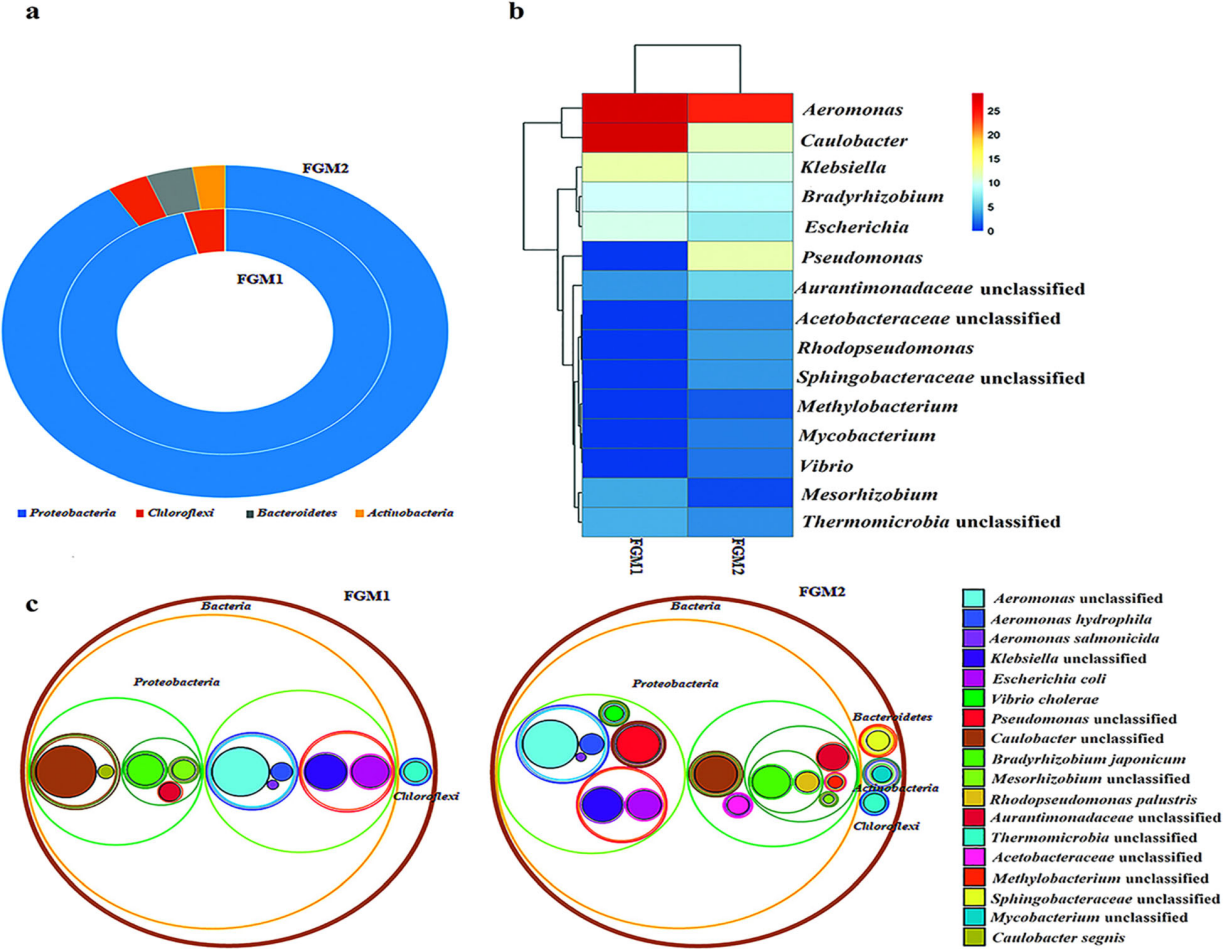
Composition of gut microbiome in endangered *Tor putitora*. **a** Figure showing relative abundance of different phyla in fish gut metagenome. **b** The hierarchical clustering in heatmap shows the abundance of different genera enriched in the gut microbial community. 7.34 and 15.27% of the reads could not be assigned to any genus in FGM1 and FGM2, respectively. **c** Species-level classification of fish gut microbiome. The diameter of the circle represents the proportion of reads assigned to the particular species. The arrangement of circles represents the hierarchy from kingdom to species level

The Illumina HiSeq 2500 platform generated a total of 284,412,950 paired-end reads, *n* = 120,716,302 paired-end reads in fish gut metagenome 1 (FGM1) and *n* = 163,696,648 paired-end reads in fish gut metagenome 2 (FGM2) yielding total 71.3 gigabase high-quality whole metagenome shotgun sequence data. The final merged read length was 200 bp. The other details regarding sequencing and assembly have been mentioned in Additional file 1: Table S1. The values of Shannon-Wiener index (H) 1.796 (FGM1) and 2.362 (FGM2) as well Simpson’s index value (1-D) of 0.7998 (FGM1) and 0.8792 (FGM2) depicted compositional shift in the microbial diversity of gut of *Tor putitora*. The rarefaction curves based on alpha diversity measures depicted the saturation phase at 1e+ 06 bp sequencing effort (Additional file 2: Figure S1). Likewise, beta diversity analysis was conducted to determine the similarity or dissimilarity in the composition of microbial communities of the samples. The Non-metric Multi-Dimensional Scaling (NMDS) plot based on the Whittaker index explained variance in the diversity of the two samples (Additional file 2: Figure S2). Similarly, pairwise correlations computed using Pearson’s method (*R*^2^ = 0.7947) depicted a positive correlation between microbial communities of two metagenomes. The microbial community was dominated by bacteria (97.07%), whereas the abundance of viruses (0.37%) and archaea (0.02%) was relatively low. Within bacteria, FGM1 and FGM2 were dominated by 95.96% and 91.33% *Proteobacteria*, respectively. Besides, *Chloroflexi*, *Actinobacteria,* and *Bacteroidetes* were also present (Fig. 1a). The relative abundance at genera level depicted that FGM1 was dominated by *Caulobacter* (28.62%), *Aeromonas* (28.18%), *Klebsiella* (12.4%), *Escherichia* (10.1%), *Bradyrhizobium* (9.55%) and *Mesorhizobium* (3.81%). Likewise, FGM2 microbial community comprised *Aeromonas* (24.51%), *Pseudomonas* (12.06%), *Caulobacter* (11.59%), *Klebsiella* (10.08%), *Bradyrhizobium* (9.12%), *Escherichia* (7.34%) and *Mesorhizobium* (0.68%). Moreover, *Rhodopseudomonas, Mycobacterium*, *Vibrio,* and *Methylobacterium* were also detected (Fig. 1b). Members of *Aurantimonadaceae*, *Acetobacteraceae,* and *Sphingobacteriaceae* could not be classified to the genus. The relative abundance of different species is illustrated in Fig. 1c.

### Functional characterization of gut microbial communities

**Fig. 2:**
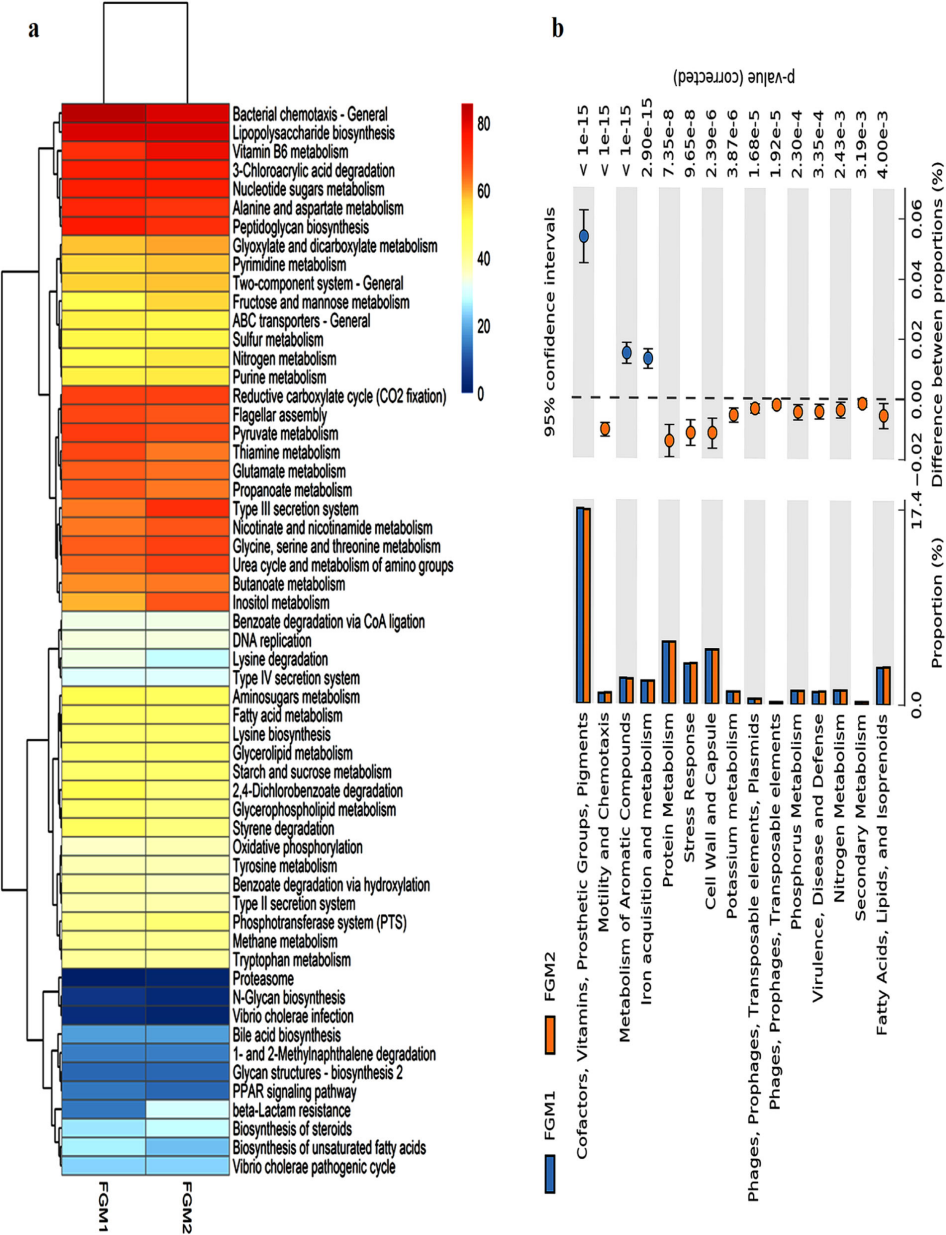
Functional analysis of metagenome depicting the major pathways enriched in the fish gut microbiome. **a** KEGG pathway analysis using Pearson’s correlation and hierarchical clustering and **b** SEED analysis using Fisher’s exact test and 0.95 confidence interval

In this study, both the KEGG and SEED databases were used to predict the metabolic potential of the gut microbiome. The KEGG pathway analysis using Pearson’s correlation and hierarchical clustering showed a higher relative abundance of pathways involved in metabolism (28.7% in FGM1, 28.9% in FGM2), followed by biosynthetic pathways (15.6% in FGM1, 12.06% in FGM2) and pathways for degradation (12.22% in FGM1 and 11.66% in FGM2). In the pathways for metabolism, vitamin B6 metabolism, nucleotide sugars, and alanine and aspartate metabolism were dominant. Under the category for biosynthesis, pathways for lipopolysaccharide biosynthesis, peptidoglycan biosynthesis, and antibiotic production were abundant. Discounting core metabolism and energy, several pathogenesis-related genetic traits, including bacterial chemotaxis, flagellar assembly, and type III secretion system, were also present (Fig. 2a). Moreover, pathways of type II- and type IV secretion systems, β-lactam resistance, and *Vibrio cholerae* pathogenic cycle was also detected (Fig. 2a). The functional assignment of normalized reads in the metagenomes with SEED database also revealed similar patterns with maximum reads assigned to the cofactors, vitamins, prosthetic groups, pigments, protein metabolism, fatty acids, lipids, and isoprenoids and metabolism of aromatic compounds. The pathways for cell wall and capsule formation, iron acquisition and metabolism, virulence, motility and chemotaxis, phages, prophages, transposable elements, plasmids showed high relative abundance in both the metagenomes. All these pathways were significantly different (Fisher’s exact test at 0.95 confidence interval, *p*-value < 0.05) among the metagenomes (Fig. 2b).

### Detection of virulence genes in the fish gut microbiome

**Fig. 3:**
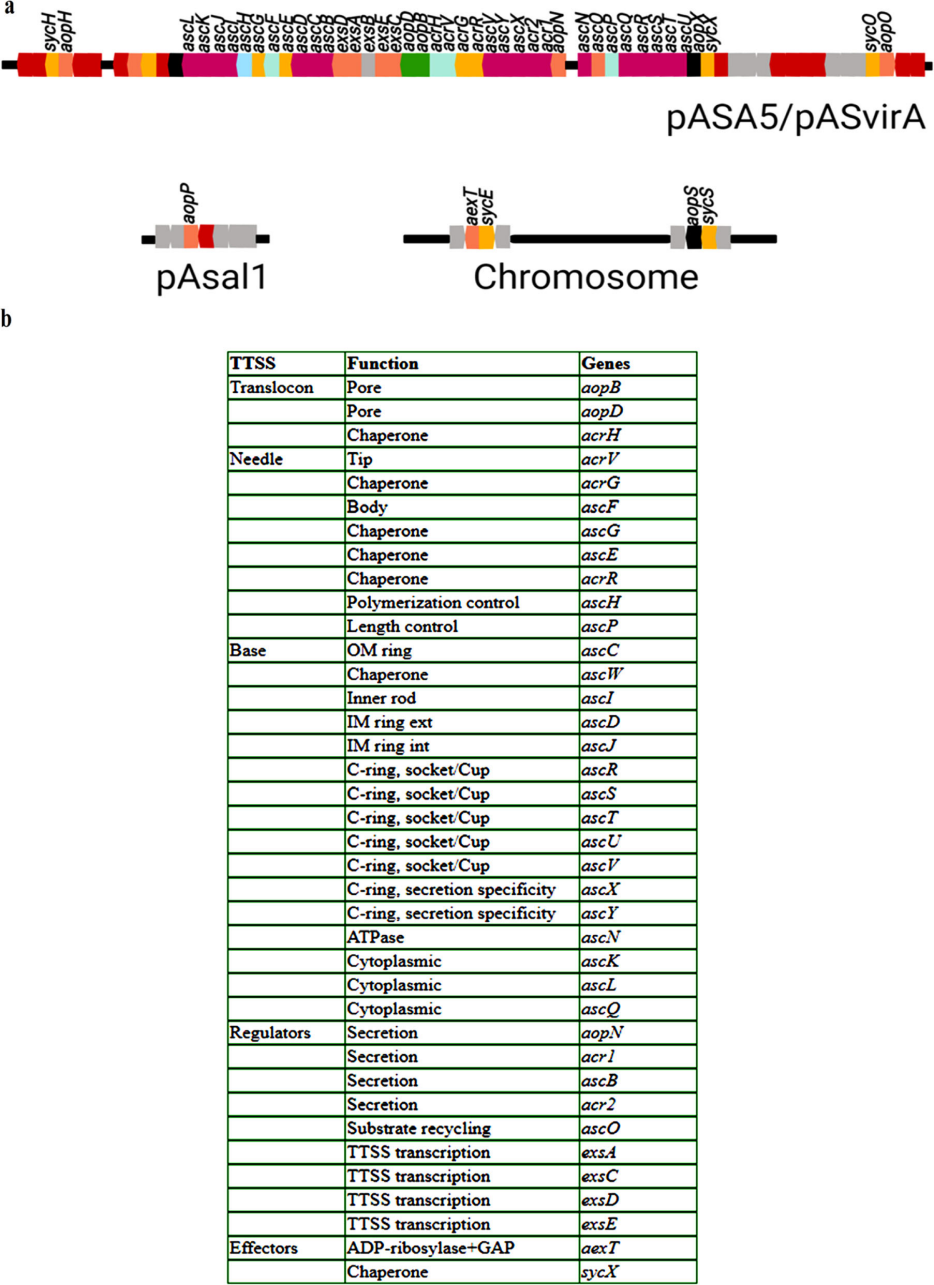
Type III secretion system in *Aeromonas salmonicida***. a** Genetic organization of type III secretion system (TTSS) genes in *Aeromonas salmonicida*. TTSS of *Aeromonas salmonicida* is located on a large plasmid pASA5 and gene encoding effector protein (*aexT*) is located on the chromosome. At stringent criteria, maximum genes of TTSS were detected while *ascD, ascE, ascF, ascK, exsB, ascP, and aopX,* and *aexT* were detected below the cut off. **b** Genes orthologous of type III secretion system of *Aeromonas salmonicida* enriched in the fish gut metagenome. Figure modified from: [51]

Fish gut microbiome could be a possible reservoir of virulence genes and resistance determinants; therefore, the gut metagenome was explored for genes encoding virulence factors of putative pathogens in fish. Search against *Aeromonas* virulence factor database depicted the presence of a total of 157 virulence genes. The key virulence factor of *Aeromonas salmonicida* is type III secretion system (TTSS) which is present on a large-sized plasmid encoded by different genes arranged in five major polycistronic operons (i) *exsA,D,ascB,C,D,E,F,G,H,I,J,K,L* (ii) *exsC,E,B* (iii) *aopN,acr1,2,ascX,Y,V,acrR,G,V,H,aopB,D* (iv) *ascN,O,P,Q,R,S,T,U* and (v) *aopX,sycX* (Fig. 3a). The Blastp searches against the virulence factor database were performed using the similarity criteria of e-value < 1e-5, percent identity ≥ 80%, alignment length/subject length ≥ 0.8, and alignment length/query length ≥ 0.8. Thirty-one genes of a total of 38 genes comprising the TTSS operon of *Aeromonas salmonicida* were detected (Fig. 3b, Additional file 1: Table S2). Some TTSS genes (*ascD, ascE, ascF, ascK, exsB, ascP, and aopX*), including *aexT,* which encodes TTSS effector protein, were detected below the cut-off. Furthermore, genes encoding hemolysin III and thermostable hemolysin in *Aeromonas veronii,* flagellar apparatus in *Aeromonas salmonicida,* and *Aeromonas hydrophila,* and HcpA, effector protein of type VI secretion system in *Aeromonas hydrophila* were also detected using the same parameters as mentioned above (Additional file 1: Table S2).

**Fig. 4:**
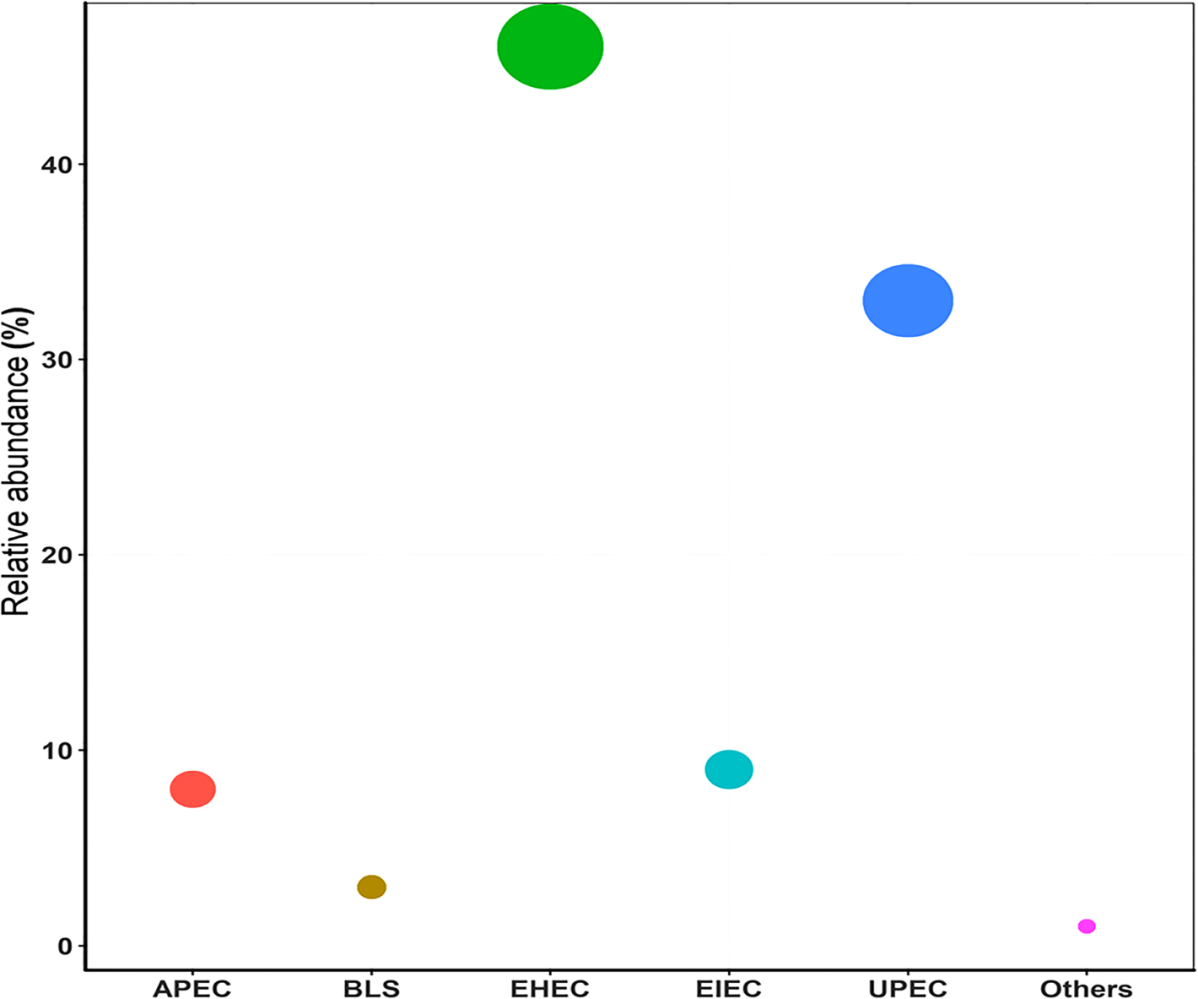
Bubble plot showing distribution of different entero-virulent *Escherichia coli* pathotypes in the fish gut ascertained on the basis of virulence genes associated with each strain. The diameter of the circle represents the relative proportion (%) of the particular *Escherichia coli* strains depending upon the relative abundance of virulence genes associated with them

Similarly, the search against the *Escherichia coli* virulence factor database depicted the presence of different virulence genes. With e-value < 1e-5, percent identity ≥ 80%, alignment length/subject length ≥ 0.8 and alignment length/query length ≥ 0.8, total 100 genes belonging to different classes of entero-virulent *Escherichia coli* strains were identified (Additional file 1: Table S3). The relative abundance of virulence genes representing enterohemorrhagic *Escherichia coli* (EHEC) strains was 46%, followed by 33% uropathogenic *Escherichia coli* (UPEC). The virulence genes associated with enteroinvasive *Escherichia coli* (EIEC) (9%), avian pathogenic *Escherichia coli* (APEC) (8%), and benign laboratory strain *Escherichia coli* (BLS) (3%) were also detected (Fig. 4). Of the total EHEC detected in the metagenomes, the relative abundance of virulence genes associated with enterohemorrhagic *Escherichia coli* O157:H7 serotype was abundant, whereas those associated with *Escherichia coli* strain RS218 were least.

The mining of *Pseudomonas* virulence genes in the fish gut metagenome resulted in the identification of 79 genes at the filter parameters. Among these, the genes for virulence factors related to adherence (45.57%) and antiphagocytosis (27.85%) were abundant. The major genes for adherence included those related to flagellar apparatus, lipopolysaccharide, and type IV pili, and antiphagocytosis was composed of genes for mucoid exopolysaccharide (alginate). Apart from these, genes for TTSS and HSI-I (Hcp1 Secretion Island I) secretion systems were also present. Also, genes encoding for exolysin (*exlA*) and iron uptake (*pvdA*, *pvdS*) were detected (Additional file 1: Table S4, Additional file 2: Figure S3).

### Distribution of antibiotic resistome

**Fig. 5:**
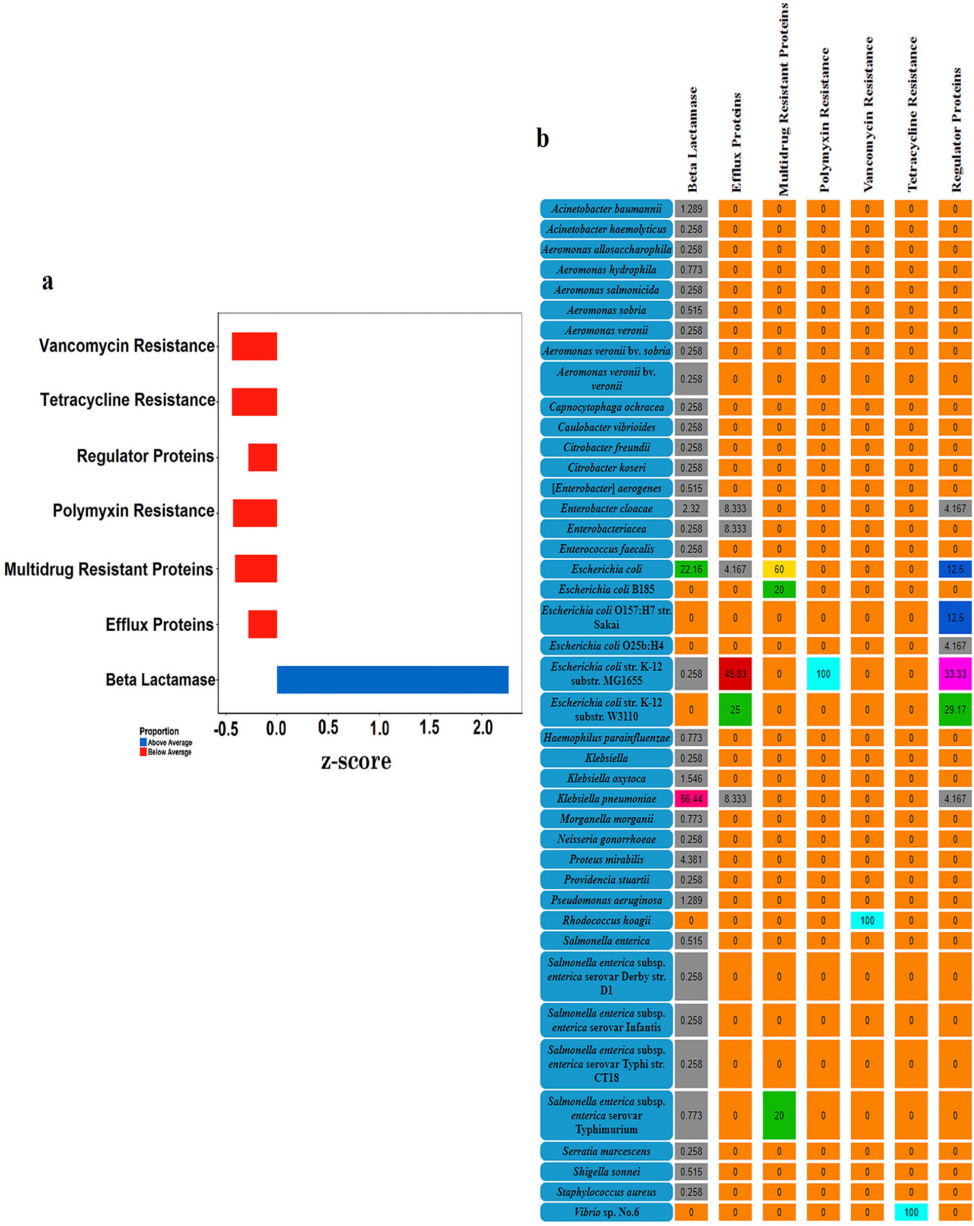
Antibiotic resistance pattern of gut microbiome in endangered *Tor putitora*. **a** Distribution of different antibiotic resistance genes in the fish gut microbiome. The data was normalized and the calculation of *z-*score showed the highly abundant genes for different variants of extended spectrum β-lactamases. **b** The relative distribution of antibiotic resistance genes in different bacteria present in the gut of *Tor putitora*. The colour code indicates the distribution of ARGs ranging from none to 100%. All the zero values are given in orange. The percentages varying from 0 to 10% are highlighted grey. The 10–20% interval is shaded blue while 20–30% range is represented by green colour. The magenta, dark-red, dark-pink and yellow colour code represent 30–40%, 40–50%, 50–60% and 60–70% distribution, respectively whereas turquoise highlights 100% abundance

Fish are associated with the dissemination of antimicrobial resistance genes (ARGs). Investigation of antibiotic resistance revealed the presence of a total of 445 ARGs. Primarily, the identified ARGs encoded for β-lactamases (87.19%), efflux pumps (5.39%), multidrug transporters (1.12%), polymyxins (0.45%), vancomycin (0.22%) and tetracycline (0.22%) resistance (Fig. 5a). In addition, different transcriptional regulators (EvgA, EvgS, GadW) involved in the controlled expression of ARGs were also identified. The relative abundance of β-lactamase was highest in *Klebsiella pneumoniae* (56.44%) trailed by *Escherichia coli* (22.42%), *Proteus mirabilis* (4.38%) and *Enterobacter cloacae* (2.32%). Contrarily, the relative abundance of efflux pumps was higher in *Escherichia coli* (75%), *Klebsiella pneumoniae* (8.33%), and *Enterobacter cloacae* (8.33%) (Fig. 5b). Within β-lactamase, different variants of extended spectrum β-lactamase (ESBL) such as *SHV*, *TEM*, *OKP,* and *CTX-M* were predominantly associated with *Klebsiella pneumoniae* (Additional file 1: Table S5).

### Reconstruction of the microbial genome and comparative genomics

**Fig. 6:**
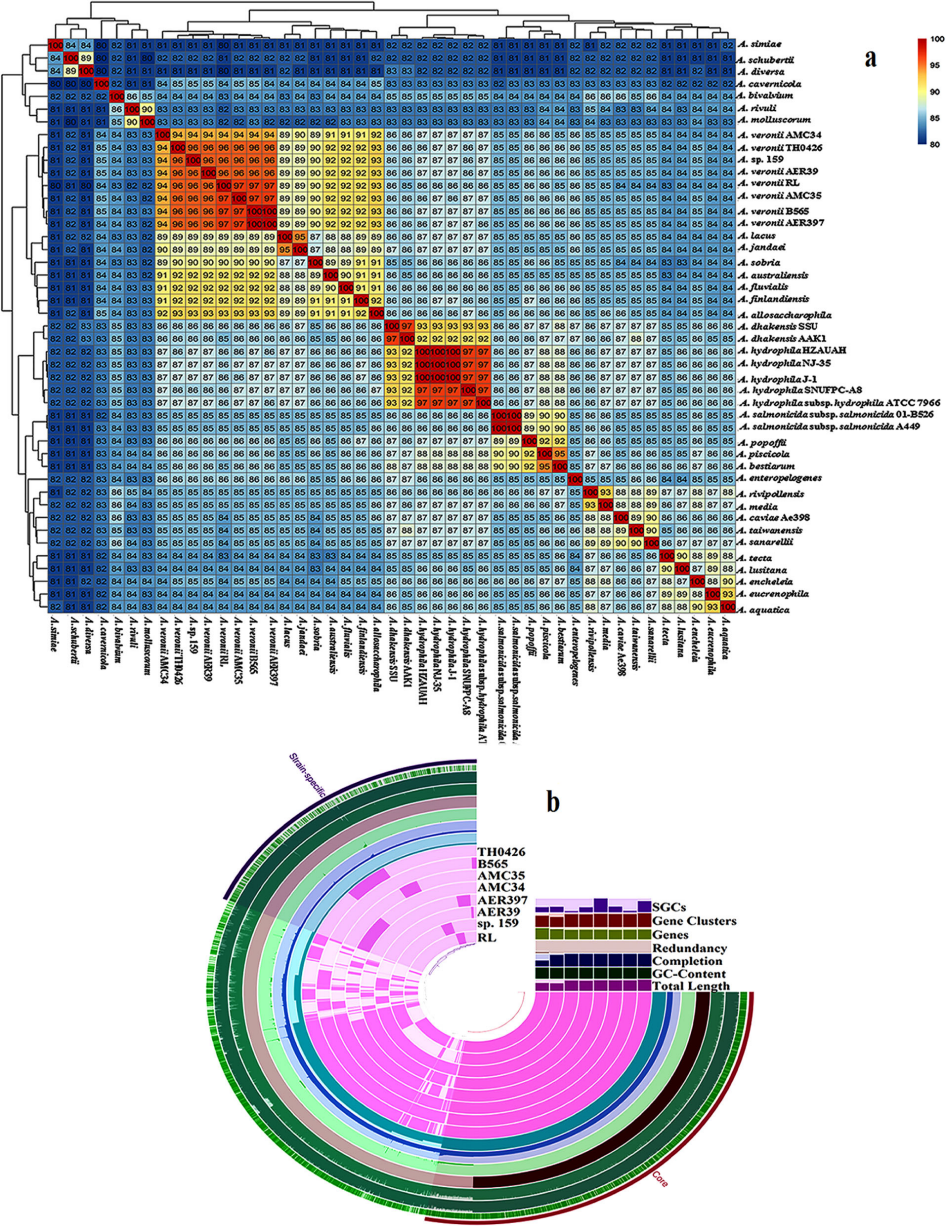
Comparative genome analysis of *Aeromonas veronii* strain RL. **a** Phylogenetic relatedness of *Aeromonas veronii* strain RL based on the average nucleotide identity values. **b** Distribution of gene clusters (core and strain-specific content) in *Aeromonas veronii* strain RL and its other closely related strains. The diagram is based on presence and absence of genes using Euclidean distance and Ward linkage. The strains’ name are used to represent the genotypes for different strains of *Aeromonas veronii*, RL – *Aeromonas veronii* RL, sp. 159 – *Aeromonas* sp. 159, AER39 – *Aeromonas veronii* AER39, AER397 – *Aeromonas veronii* AER397, AMC34 – *Aeromonas veronii* AMC34, AMC35 – *Aeromonas veronii* AMC35, B565 – *Aeromonas veronii* B565, TH0426 – *Aeromonas veronii* TH0426

In this study, we reconstructed a microbial genome of 3.5 Mb. RAST analysis (RAST Id: 6666666.356227) depicted that reconstructed microbial genome of 3,501,394 bp size consisted of 3464 coding sequences and 383 subsystems and showed close resemblance with *Aeromonas veronii* B565, *Aeromonas hydrophila* subsp. *hydrophilaATCC* 7966, and *Aeromonas salmonicida* subsp. *salmonicida* A449. To further gain insight into the precise phylogenetic relatedness, whole genome-based average nucleotide identity (ANI) analysis was performed using publicly available representative genomes of *Aeromonas* species. Estimates of ANI values (> 94%) showed that reconstructed microbial genome clustered together with strains of *Aeromonas veronii* (Fig. 6a, Additional file 1: Table S6). Henceforth, it was designated as *Aeromonas veronii* strain RL.

The pathogenicity of *Aeromonas* species is linked to different virulence factors, including adhesion proteins, siderophore secretion, toxins, and lipopolysaccharides (LPSs). In order to confirm the pathogenic properties of strain RL, we performed its comparative genomic analyses with reported pathogenic strains of *Aeromonas veronii* and an uncharacterized *Aeromonas* sp. 159, which was phylogenetically closer to selected *Aeromonas veronii* strains*.* A close look at the number of orthologous genes revealed the presence of 1627 single-copy genes (core) determined using the GET_HOMOLOGUES pipeline (Fig. 6b). Genes encoding different virulence factors including LPS biosynthetic gene cluster, hemolysin, flagellar biosynthesis and assembly, siderophore secretion, two-component system, type I, and type II proteins, type IV pilus genes, outer membrane porin proteins, proteases were conserved in *Aeromonas veronii* genomes. In addition, genes encoding efflux pumps, β-lactamase, multidrug resistance proteins, dihydrofolate reductase, resistance-nodulation-cell division (RND) multidrug efflux transporters and major facilitator superfamily (MFS) were also present in the core genome. Interestingly, quorum-sensing regulator protein, which is directly involved in the pathogenesis, was found in the core (Additional file 1: Table S7). Strain RL also harboured genes encoding different virulence factors viz. lipase, collagenase, hemolysin, phospholipase C, and serine protease. In addition, strain RL and *Aeromonas veronii* TH0426 harboured L-serine dehydratase, which was earlier considered unique to hypervirulent strains of *Aeromonas hydrophila* (Table 1, Additional file 1: Table S8).

**Table 1:** Presence and absence matrix of different virulence factors among known pathogenic strains of *Aeromonas* species

Virulence factors	*A. veronii* RL	*A. veronii* B565	*A. veronii* TH0426	*A. hydrophila* ATCC 7966	*A. hydrophila* HZAUAH	*A*. *hydrophila* J1	*A*. *hydrophila* NJ 35	*A. salmonicida* A449
Lipase	+	+	+	+	+	+	+	+
Serine Protease	+	+	+	+	+	+	+	+
Collagenase	+	+	+	+	+	+	+	+
Phospholipase C	+	+	+	+	+	+	+	+
Metalloprotease	+	+	+	+	+	+	+	+
L-serine dehydratase	+	–	+	+	+	+	+	–
Oligopeptidase A	+	+	+	+	+	+	+	+
Sensor histidine protein kinase	+	+	+	+	+	+	+	+
Hemolysin	+	+	+	+	+	+	+	+
Thermostable hemolysin	+	+	+	+	+	+	+	+
Cytolysin and hemolysin (Hly A)	–	–	–	–	+	+	+	+

## Discussion

The values of the Shannon-Wiener index (H) were in the range of 1.8–2.4, and Simpson’s index value (1-D) varied from 0.8–0.9, which was consistent with values reported by other studies [52–54] indicating considerable sample diversity in the metagenomes. The composition of gut microbiota has been explored in different fish species as a function of dietary changes, the impact of host genotype, and different environmental factors [1, 2, 55–58]. The beta diversity analysis also showed variations from site 1 to site 2, mainly because microbiome composition can be influenced by the environment [58]. Studies have confirmed the influence of gut microbiota in host development, physiology, and health maintenance [22, 55, 59]. Therefore, a comprehensive analysis of the taxonomic composition and function of gut microbiota of endangered *Tor putitora* is crucial for understanding the influence of gut microflora on the host. To our best knowledge, the present study is the first report showing shotgun metagenomic analysis of microbial communities present in the gut of an endangered freshwater fish. In this study, *Proteobacteria* were dominantly present in the intestinal tract of *Tor putitora,* which conforms to previous studies including Prussian carp, grass carp, crucian carp, bighead carp, and *Labeo rohita* (rohu) [1, 2, 19, 57, 60, 61]. Studies have also shown the presence of *Chloroflexi*, *Actinobacteria*, *Firmicutes*, *Fusobacteria,* and *Bacteroidetes* in the intestinal tract of different carp species [7, 57, 58, 60–64].

Fish are in continuous contact with the complex and dynamic planktonic microbiota; therefore, it is expected that the gut microbiota in fish is largely affected by microbes in the environment [65]. The dominant microbiota in the intestinal contents of carps included bacteria from families *Caulobacteraceae,* among others [66]. Also, the prey of different fish species in the Chany (eutrophic) lake showed relatively higher abundances of bacteria from family *Caulobacteraceae* [66]. The high relative abundance of genus *Caulobacter* in the gut of *Tor putitora* could be attributed to its feeding on insects, macrophytes, rotifers, and small fish [45]. The intestine of healthy juvenile salmon had *Caulobacter* as one of their main bacterial components [67]. The occurrence of *Caulobacter* spp. on the eggs of *Gadus morhua* L. and *Hippoglossus hippoglossus* indicated that eggs were colonized by bacteria before spawning and could be because of preinvasion from the gut [68]. Alongside resident autochthonous microbes, the fish gut is considered as the principal reservoir of *Aeromonas*, *Pseudomonas*, *Vibrio*, *Streptococcus*, *Mycobacterium,* and other coliforms [26–30, 41]. The presence of *Escherichia coli* in different tissues and organs of the fish indicates the bacteriological conditions of the water inhabited by the fish [29]. In Gobindsagar lake, which was our sampling site, industrial and domestic effluents are discharged into the water body, it is also used for bathing and recreational activities, thereby deteriorating the quality of water [69, 70]. Thus, pathogenic microbes from such polluted environments can be transmitted to fish, thereby colonizing their guts [26]. Moreover, the relatively high prevalence of *E. coli* in the gut could be because they multiply rapidly when the temperature is between 16 °C and 20 °C [29] and in the present study, the water temperature was 18.033 ± 0.153 °C at sampling site 1 and 20.467 ± 0.451 °C at sampling site 2 (Additional file 1: Table S9). Also, they have long retention periods suggesting that they could carry bacteria in their digestive tract to non-polluted water [29]. *Klebsiella pneumoniae* is an opportunistic pathogen responsible for causing nosocomial infections and are found in the gastrointestinal tract of the host [71, 72]. Again, the presence of *K. pneumoniae* in the gut may relate to the water environment, and its presence has been reported from tilapia, rohu [19, 73]. Being an opportunistic pathogen, it may cause disease under favorable conditions, as was the case in Maldive’s clown fish when the concentration of un-ionized ammonia increased in the culture tank [74]. *Pseudomonas* species are another group of bacterial communities that are frequently associated with fish as one of the dominant microbes and have been isolated from their skin, gills and intestine [13, 35, 75–77]. *Pseudomonas* might aid in digestion by the production of proteases and along with *Photobacterium* spp. might produce chitinases [75]. Also, these species have been evaluated as potential probiotics in aquaculture [78–80], and their live inoculum can be used to mitigate oomycete diseases [81]. Tripathy et al. (2007) proposed that they are widespread and numerous, hence may act as secondary invaders of fish compromised with pathogens and may become involved in the disease processes [39, 76]. Thus, there is a very blurred boundary between pathogens and commensals [82].

Human beings have used large amounts of antibiotics as growth factors in aquaculture, agriculture, and livestock, resulting in the contamination of the immediate environment [83]. The pathogens then exploit community changes induced by antibiotics, wherein microbiome serves as a reservoir of virulence factors and resistance determinants [82]. Given that many virulence genes are coded in extra-chromosomal elements, the horizontal transfer of such genes to other non-pathogenic species might occur by genetic elements of varying mobility such as plasmids and transposable elements [84, 85]. The functional annotation of gut metagenome revealed the presence of different mobile genetic elements that might aid in the transfer of virulence properties to harmless strains rendering them potential pathogens [83]. Also, it has been stated that virulence factors are needed in bacteria’s “struggle for existence” against microscopic adversaries [82]. For example, enterohemorrhagic serotype *Escherichia coli* O157:H7 (EHEC) is a commensal in the bovine gut, but acts as a pathogen for humans [82]. Therefore, it could be concluded that the presence of virulence factors in the fish gut metagenome may not be an indication of unhealthy gut microbiota but can serve as a reservoir for dissemination of genes to mutualistic or commensal bacteria which can pose a serious threat to fish and public health by their virtue of being converted into potential pathogens. Escudeiro et al. (2019) proposed that there exists a correlation between antibiotic resistance determinants and virulence factors diversity in metagenomes and speculated that by selecting for resistant bacteria, we may end up selecting for more virulent strains as a side effect of antibiotics usage [83]. Antibiotic resistance genes have been frequently reported in isolates belonging to the families *Pseudomonadaceae*, *Enterobacteriaceae,* and *Rhizobiaceae* [84]. Besides, multidrug-resistant strains of *Escherichia coli* and *Klebsiella pneumoniae* have been reported from wild and commercial fish and other seafood [43, 86–88]. The extended-spectrum β-lactamase (ESBL) was found to be predominantly associated with *Klebsiella pneumoniae* present in the gut of *Tor putitora*. Similar to this study, genes encoding extended spectrum beta-lactamase (*TEM*, *CTX-M-1*) and multidrug resistance proteins (*mdtA*, *mdtB*, *mdtC*) were also found in the gut microbiome of freshwater Indian carp [19].

Members of *Aeromonas* have been reported from fish with a wide range of both beneficial and pathogenic outcomes [75, 89, 90]. The furunculosis committee had considered the fish intestine as an important niche for the isolation of *Aeromonad* species [35, 91–93]. In this study, the prominently detected *Aeromonas* species were *Aeromonas hydrophila*, *Aeromonas salmonicida,* and *Aeromonas veronii.* Several virulence factors have been characterized for *Aeromonas* species, and the type III secretion system (TTSS), which can efficiently inject anti-host virulence determinants (toxins) into the host cells, is main virulent factor reported in strains of *Aeromonas salmonicida* and *Aeromonas hydrophila* [51, 94–97]. Functional investigation showed the presence of different virulence genes of *Aeromonas hydrophila*, and complete operon encoding TTSS of *Aeromonas salmonicida* (Fig. 3b, Additional file 1: Table S2). The role of genes encoding flagellar apparatus, and bacterial chemotaxis has been well elucidated in *Helicobacter pylori*, *Pseudomonas aeruginosa*, *Vibrio cholerae* allowing them to colonize and invade the host’s mucosa [98]. Apart from these, type IV pili serve as important structures for adhesion to epithelial cells and are involved in biofilm formation and twitching motility [98]. These observations suggest the presence of virulent strains of *Aeromonas* in the gut of *Tor putitora,* indicating that intestine may be a possible route of infection. Moreover, the reconstruction of *Aeromonas veronii* strain RL genome reflected its abundance*. Aeromonas veronii* strains have been conspicuously detected in the intestine of fish [30, 99]. Recently, *Aeromonas jandaei* and *Aeromonas veronii* were reported as the disease causative agent and linked to mortality in Nile tilapia [91]. Core genome analysis highlighted the virulent properties of strain RL and showed the conservation of virulence genes. Comparative genome analysis confirmed the virulent properties of strain RL genome, which harbored several virulence genes encoding lipase, collagenase, hemolysin, thermostable hemolysin, phospholipase C and serine protease which are well-characterized virulence factors of *Aeromonas* species [94]. Intriguingly, strain RL possessed a gene encoding L-serine dehydratase, which was earlier considered to be uniquely present in hypervirulent strains of *Aeromonas hydrophila*. The role of L-serine dehydratase in the colonization of the avian gut by *Campylobacter jejuni* has been reported [100]. The presence of virulent properties does not reflect that they are actual pathogens; however, under conditions of stress-induced infections, the gut may be the primary location for colonization by *Aeromonas*. Clearly, additional studies are needed to understand the role of *Aeromonas* species in the gut of *Tor putitora*.

Previous studies had shown that intestinal microbiota of freshwater fish is dominated by phyla such as *Proteobacteria*, *Fusobacteria*, *Firmicutes,* and *Bacteroidetes* whose members are able to decompose plant polymers and ferment organic compounds providing nutrients and energy to fish [7, 35, 58, 60, 62, 101, 102]. *Aeromonas* species, representative of *Gammaproteobacteria,* are well-characterized fish pathogens, but beneficial cellulose-degrading *Aeromonas* species had also been cultured from the intestinal tract of grass carp [2]. Likewise, *Cetobacterium* (*Fusobacteria*), which can produce vitamin B_12_ were abundantly present in the intestinal tract of *Cyprinus caprio* and *Tor tambroides* [7, 103, 104]. Interestingly, occurrences of symbiotic bacteria such as *Bradyrhizobium* and *Mesorhizobium*, which are generally associated with plant roots, reflect the omnivorous feeding habit of *Tor putitora* [45]. The diverse microbial community present in the gut of *Tor putitora* comprised of both non-pathogenic (symbiotic or beneficial) microbes and putative pathogens. The diverse intestinal communities are more beneficial for their host and stable to environmental disturbances [65]. Consistent with this statement, higher alpha diversity was frequently detected in the healthy fish compared to diseased fish [65], and measures of alpha diversity in *Tor putitora* were comparable to those in healthy fish in other studies [65]. The environmental factors adversely affecting the non-pathogenic community (microbial dysbiosis) would possibly decrease the competition to gut pathogens allowing their colonization and proliferation in the gut of *Tor putitora*. Also, the risk involved in the consumption of contaminated fish may not necessarily be associated with bacteria present in the edible tissues, but infection may also occur during handling of the fish, cross-contamination to other food sources is likely to occur when the fish is prepared and cleaned for consumption. Also, the presence of antibiotic-resistant strains in the gut poses a threat to public health by their virtue of being consumers.

## Conclusions

The present study is a pioneer attempt to investigate the microbial diversity and functional potential of gut microbial communities of endangered fish *Tor putitora*. This fish species is vital for ecological and ecosystem stability; thus, potential mobilization of antibiotic resistance genes through gut microbes has serious implications both for the fish and human health. This baseline data on gut microbiome clears that the microbiota affects its host in more than one way, and this study is thought to bring a plenitude of understanding of their functional potential in the host and expand current notions of the fish gut microbiome.

## Methods

### Site selection and sampling

**Fig. 7:**
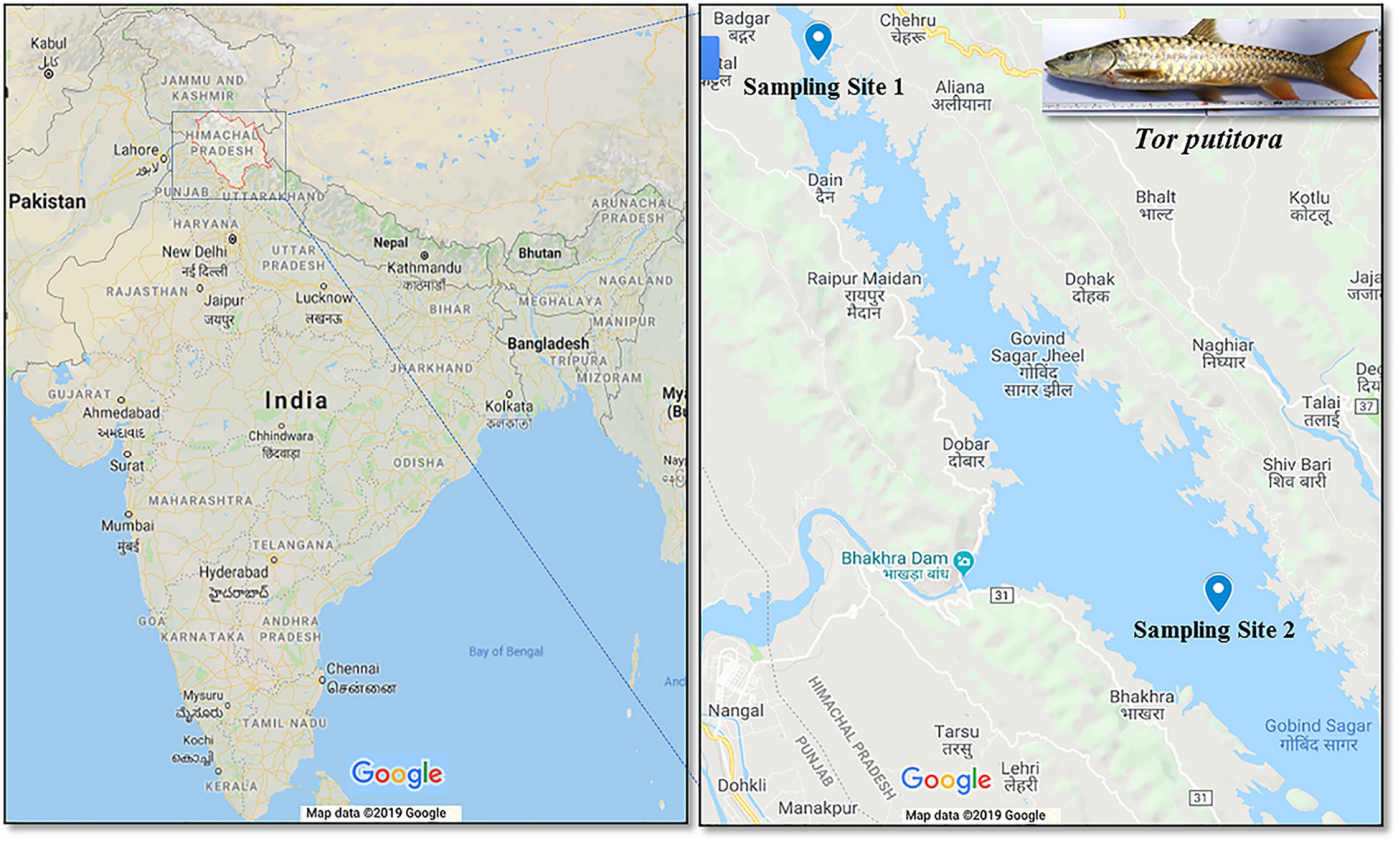
The map of sampling location indicating the two points of collection of fish samples from Gobindsagar reservoir, Himachal Pradesh, India

In this study, sampling was performed from two fish landing stations located at the Gobindsagar reservoir, Himachal Pradesh, India (Fig. 7). The first sampling was done from Gulehar site (31.4047°N, 76.4968°E; lotic water system) and second sampling site was Bhakra barrage (31.5269°N, 76.3968°E; lentic water system). A total of ten healthy fish specimens ranging from 50-53 cm in length and 1650–2250 g in weight were collected from each of the two sites (Additional file 1: Table S10). Samples were brought to the laboratory in dry ice. Each fish was aseptically dissected for its whole gut contents, and samples were stored at − 80 °C. The Physico-chemical parameters of water such as temperature, pH, electrical conductivity, total dissolved solids (TDS) and dissolved oxygen (DO) were measured immediately after sampling by an Orion 5-Star Portable pH/ORP/DO/Conductivity Multimeter (Thermo Fisher Scientific Inc. [NYSE: TMO], MA, USA).

### DNA isolation, sequencing and data processing

The DNA was isolated from the whole gut contents of each fish sample using a metagenomic DNA isolation kit (PowerSoil® DNA Isolation Kit, MO BIO). The metagenomic DNA was quantified using the NanoDrop ND-1000 spectrophotometer (NanoDrop Technologies, Inc., Wilmington, DE, USA). The metagenomic DNA was visualized on 0.8% agarose gel electrophoresis. The DNA from each fish gut content sample was pooled in equimolar concentration for fish gut metagenome 1 (FGM1) and fish gut metagenome 2 (FGM2) samples representing sampling site 1 and sampling site 2, respectively [58, 63]. The whole metagenome shotgun DNA sequencing was performed at Beijing Genomics Institute (Shenzhen, China) on Illumina HiSeq 2500 platform. The raw data were processed for a quality check using the FastQC software [105]. The duplicate’s reads were removed using Picard [106], and any reads with a quality score of less than 20 were discarded.

### Taxonomic and functional characterization

Microbial diversity was analyzed on filtered reads using MetaPhlAn, which has its own database comprising of ~ 1 M unique clade-specific marker genes [21, 107]. For the classification at the species level, the relative abundances were normalized according to the median genome size of each predicted species and the number of total reads in the sample [108, 109]. The rarefaction analysis and alpha and beta diversity measures were computed in Megan6 [110]. For functional characterization, raw reads were submitted for BLAST search against the *nr* database using diamond BLASTx at e-value 1e-3, similarity > 90%, and alignment length > 20 amino acids [111]. Using the best-hit algorithm, individual reads were described to belong to a class in the particular classification system [112]. The method was as follows: For a read ‘r’, let ‘a’ describe the highest-scoring alignment to a reference protein belonging to functional class ‘c’ and the number of reads that mapped to the individual proteins were then analysed using databases eggNOG [113], KEGG [114] and SEED [115]. The data was normalized by dividing the binned read counts for each pathway with the number of total reads in the sample [109].

### Detection of virulence and antibiotic resistance genes

The de novo assembly of paired-end reads from each sample was done using IDBA-UD at different k-mers [116]. The open reading frames (ORFs) were then predicted on the assembled contigs using FragGeneScan [117]. The protein sequences encoded by virulence genes of *Aeromonas* and *Pseudomonas* were retrieved from Virulence Factors Database (VFDB) [118]. Likewise, *Escherichia coli* virulence factors (EVF) database was created using the protein sequences encoded by different virulence genes of *Escherichia coli* available at VFDB [118], and Victors Virulence Factors database [119]. Distribution of antibiotic resistance genes in fish gut metagenome was analyzed using ‘The Comprehensive Antibiotic Resistance Database’ (CARD) [42, 120, 121]. The z-score for antibiotic resistome was calculated using the mean and standard deviation of the abundances of all individual antibiotic resistance genes in the fish gut metagenome. The BLASTp searches were performed using the assembled contigs to identify the virulence genes (*Escherichia coli*, *Aeromonas* spp. and *Pseudomonas* spp.) and major ARGs. The virulence genes and ARGs which fulfilled the following similarity criteria (cut off): e-value < 1e-5, percent identity ≥ 80%, alignment length/subject length ≥ 0.8, and alignment length/query length ≥ 0.8 were included in the study [122].

### Assembly of the microbial genome and comparative genomics

The microbial genome was reconstructed from the gut metagenome sequence using Metabat2 [123, 124]. The quality and completeness of the assembled genome was checked using CheckM [125]. The reconstructed genome was annotated using Rapid Annotations using Subsystems Technology (RAST) [126]. For comparative genome analysis, all the nearest neighbours annotated in RAST, as well as the representative genotypes available at National Center for Biotechnology Information (NCBI), were included [127]. Whole genome-based ANI analysis was done, and genomes were clustered using kmeans clustering. The clustering analysis revealed the monophyletic neighbours of the metagenome assembled genome (MAG) and orthologous gene identification was performed using the GET_HOMOLOGUES pipeline [128] at the following criteria: -E value 1e-5 (expectation value), −C > 75 (percentage coverage in BLAST alignments) and -F 1.5 (inflation value).

## Supplementary information

**Additional file 1 **Supplementary tables. Supplementary **Table S1.** contains details regarding sequencing and assembly of the metagenomes FGM1 and FGM2. **Table S2**, **Table S3 and Table S4**. contain list of virulence factors of *Aeromonas* species, *Escherichia coli* pathotypes and *Pseudomonas* species identified in gut metagenome. **Table S5**. enlists the antibiotic resistome of fish gut metagenome. **Table S6**. describes the general genome features of *Aeromonas veronii* strain RL and its nearest neighbours, while **Table S7**. lists core genes conserved in the selected genomes of *Aeromonas veronii* strains. **Table S8**. is the RAST annotations of assembled genome of *Aeromonas veronii* strain RL. **Table S9**. provides an account of various physico-chemical properties of water collected from two sampling sites mentioned in **Table S10** and depicted in Fig. 7. **Table S10**. describes length and weight of the fish species collected from Gobindsagar reservoir (Gulehar and Bhakra).

**Additional file 2 **Supplementary figures. **Figure S1**. shows the rarefaction curves of samples. **Figure S2**. depicts the beta diversity estimates of the samples using Non-metric Multi-Dimensional Scaling plot based on Whittaker distance. **Figure S3**. shows the relative abundance of different virulence factors of *Pseudomonas* spp.

## Data Availability

The raw sequencing read data set and metagenome assembled microbial genome have been deposited in the European Nucleotide Archive (ENA) under the project accession number PRJEB30661, https://www.ebi.ac.uk/ena/data/search?query=PRJEB30661.

## Abbreviations

ARGs: Antibiotic Resistance Genes
FGM: Fish Gut Metagenome
TTSS: Type III Secretion System
EHEC: Enterohemorrhagic *Escherichia coli*
