# Supplementary figures and images for: Gut microbiome of endangered Tor putitora (Ham.) as a reservoir of antibiotic resistance genes and pathogens associated with fish health
Source: BMC Microbiol. 2020 Aug 12;20:249. doi: 10.1186/s12866-020-01911-7 (PMC7425606; doi:10.1186/s12866-020-01911-7)

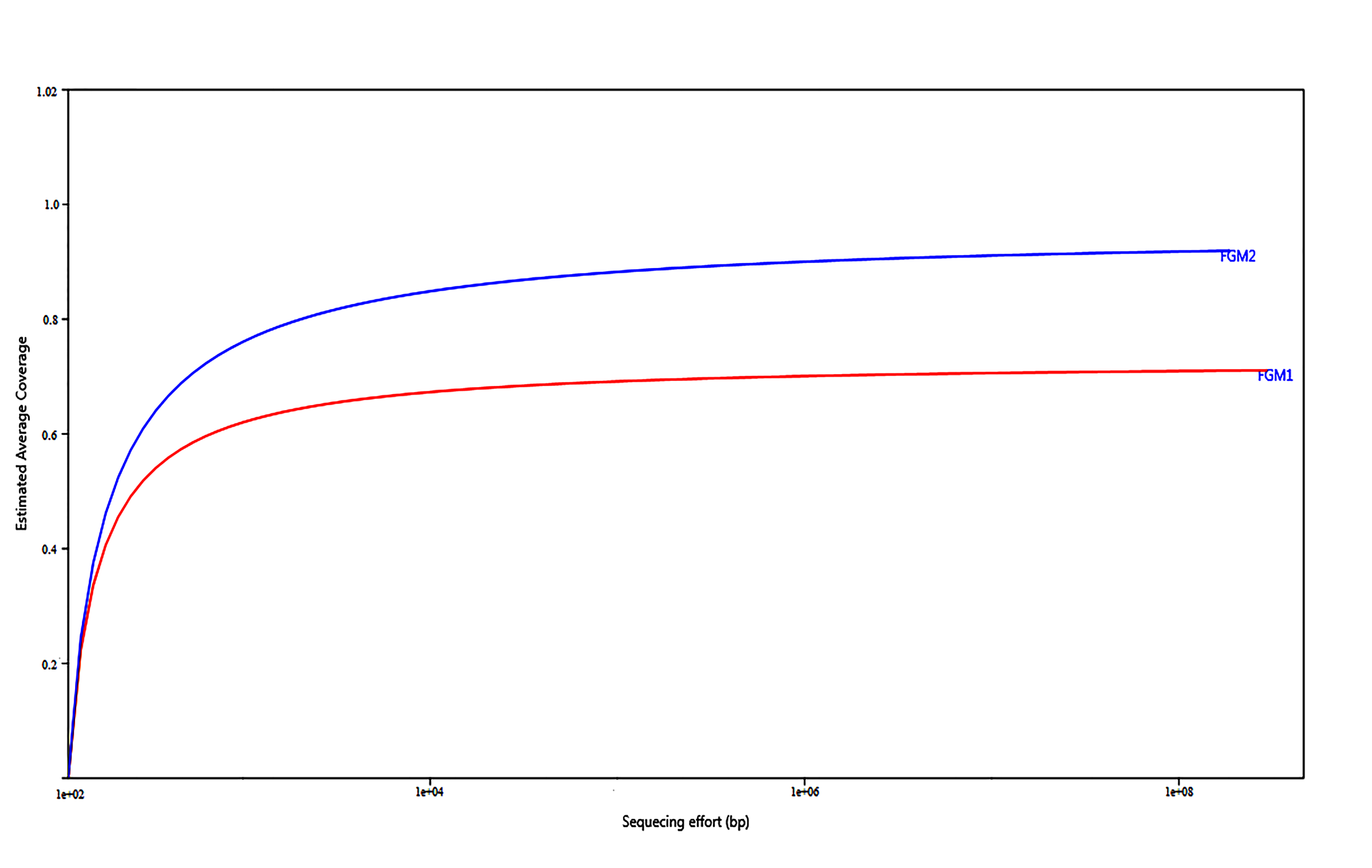


Figure S1


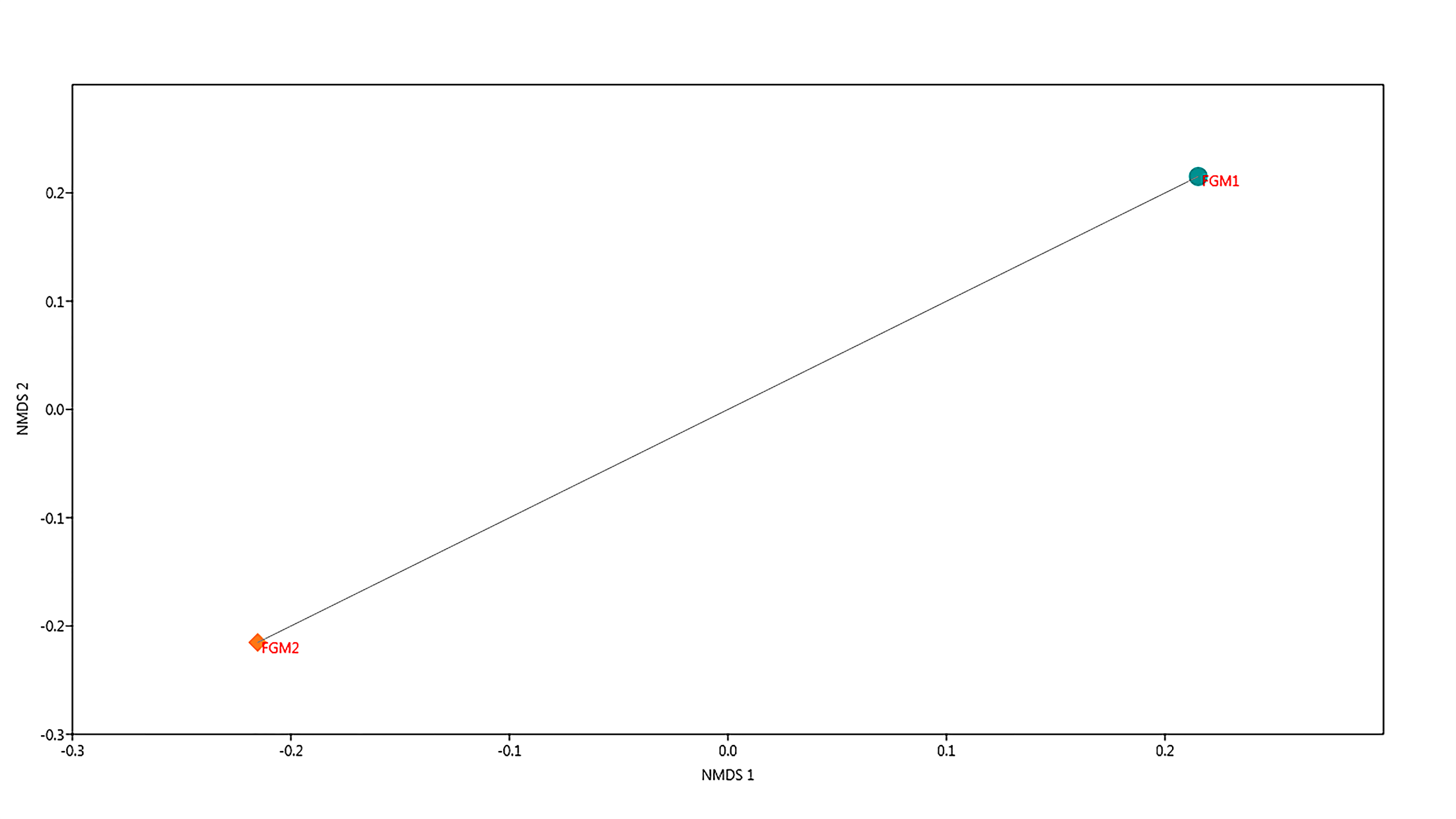


Figure S2


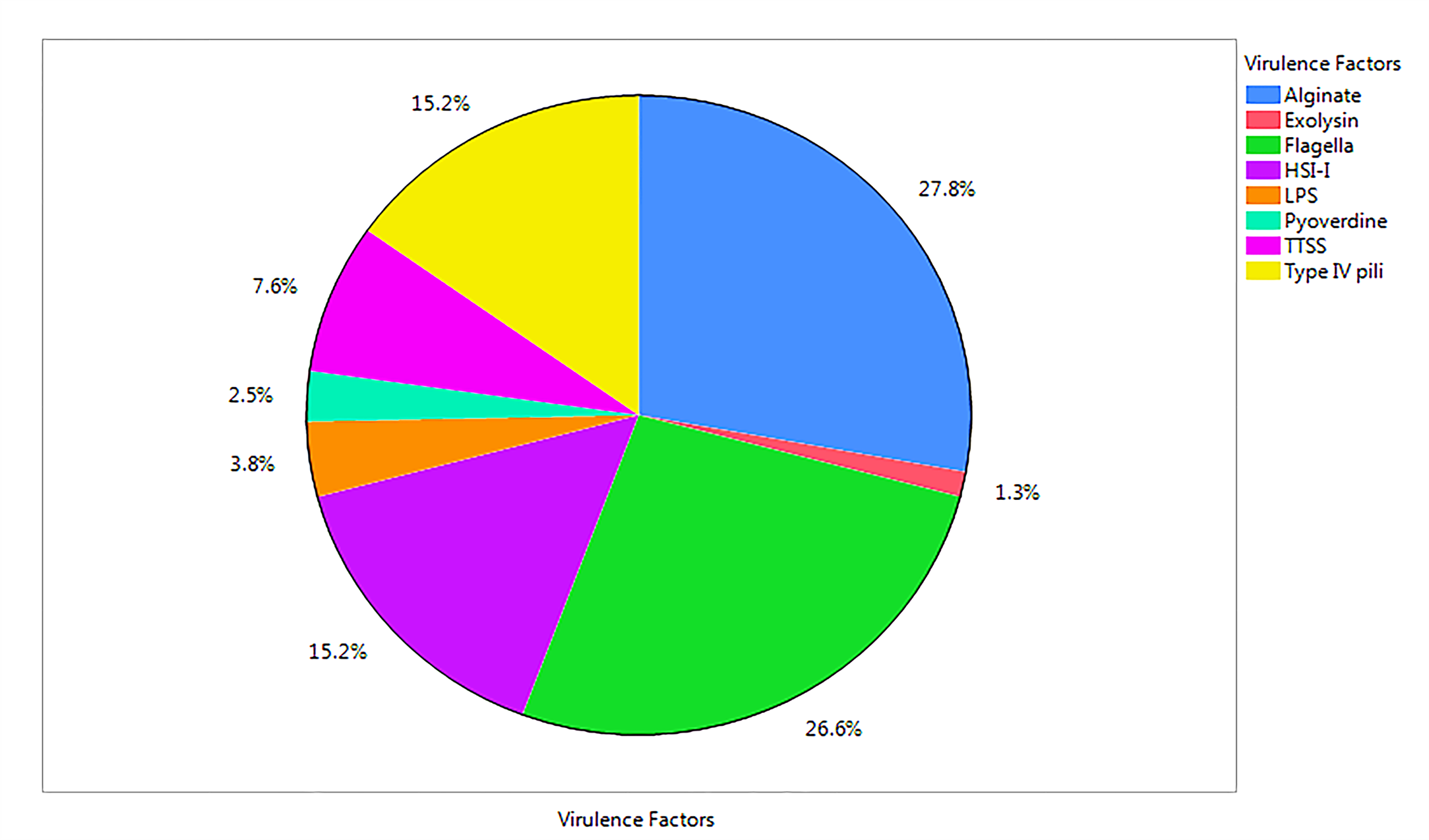


Figure S3

Supplement: Supplementary file 2 — Additional file 2 Supplementary figures. Figure S1. shows the rarefaction curves of samples. Figure S2. depicts the beta diversity estimates of the samples using Non-metric Multi-Dimensional Scaling plot based on Whittaker distance. Figure S3. shows the relative abundance of different virulence factors of Pseudomonas spp. [file 12866_2020_1911_MOESM2_ESM.docx]
